# An early and stable mouse model of polymyxin-induced acute kidney injury

**DOI:** 10.1186/s40635-024-00667-y

**Published:** 2024-10-01

**Authors:** Linqiong Liu, Yuxi Liu, Yu Xin, Yanqi Liu, Yan Gao, Kaijiang Yu, Changsong Wang

**Affiliations:** 1 Departments of Critical Care Medicine, The Fourth Affiliated Hospital of Harbin Medical University, Harbin, 150001 Heilongjiang China; 2 Departments of Critical Care Medicine, The First Affiliated Hospital of Harbin Medical University, Harbin, 150001 Heilongjiang China; 3 Heilongjiang Provincial Key Laboratory of Critical Care Medicine, 23 Postal Street, Nangang District, Harbin, 150001 Heilongjiang China

**Keywords:** Polymyxin B, Polymyxin E, AKI, Mouse model, GFR, KIM-1, NGAL

## Abstract

**Background:**

Polymyxins have been revived as a last-line therapeutic option for multi-drug resistant bacteria and continue to account for a significant proportion of global antibiotic usage. However, kidney injury is often a treatment limiting event with kidney failure rates ranging from 5 to 13%. The mechanisms underlying polymyxin-induced nephrotoxicity are currently unclear. Researches of polymyxin-associated acute kidney injury (AKI) models need to be more standardized, which is crucial for obtaining consistent and robust mechanistic results.

**Methods:**

In this study, male C57BL/6 mice received different doses of polymyxin B (PB) and polymyxin E (PE, also known as colistin) by different routes once daily (QD), twice daily (BID), and thrice daily (TID) for 3 days. We continuously monitored the glomerular filtration rate (GFR) and the AKI biomarkers, including serum creatinine (Scr), blood urea nitrogen (BUN), neutrophil gelatinase-associated lipocalin (NGAL), and kidney injury molecule-1 (KIM-1). We also performed histopathological examinations to assess the extent of kidney injury.

**Results:**

Mice receiving PB (35 mg/kg/day subcutaneously) once daily exhibited a significant decrease in GFR and a notable increase in KIM-1 two hours after the first dose. Changes in GFR and KIM-1 at 24, 48 and 72 h were consistent and demonstrated the occurrence of kidney injury. Histopathological assessments showed a positive correlation between the severity of kidney injury and the changes in GFR and KIM-1 (Spearman’s rho = 0.3167, *P* = 0.0264). The other groups of mice injected with PB and PE did not show significant changes in GFR and AKI biomarkers compared to the control group.

**Conclusion:**

The group receiving PB (35 mg/kg/day subcutaneously) once daily consistently developed AKI at 2 h after the first dose. Establishing an early and stable AKI model facilitates researches into the mechanisms of early-stage kidney injury. In addition, our results indicated that PE had less toxicity than PB and mice receiving the same dose of PB in the QD group exhibited more severe kidney injury than the BID and TID groups.

**Supplementary Information:**

The online version contains supplementary material available at 10.1186/s40635-024-00667-y.

## Background

Acute kidney injury (AKI) is a clinically severe condition with high incidence and mortality. Globally, the incidence of AKI even may be up to 66%, with the mortality rate of 20% in non-ICU hospitalized patients and 40–80% in intensive care setting [1, 2]. In addition, drug-induced nephrotoxicity plays a significant role in the occurrence of AKI, where antibiotic-induced nephrotoxicity accounting for up to 36% [3, 4]. Increasing antibiotic resistance in Gram-negative bacteria (GNB) is a major cause of the significant increase in global morbidity and mortality. This resistance has emerged against almost all clinically used antibiotics except polymyxins [1]. Therefore, polymyxins will remain the last line of treatment for resistant Gram-negative infections and retain a significant portion of global use due to their residual antibiotic activity, worldwide availability and cost [5, 6].

The incidence of polymyxin-induced AKI can be as high as 60% [7], with kidney failure occurring from 5 to 13% of cases [8]. Therefore, nephrotoxicity severely limits the clinical use of polymyxins. Moreover, previous studies have shown that polymyxin E (PE, a.k.a. colistin) is associated with a higher risk and earlier occurrence of AKI than PB [9]. Conversely, some studies have shown that the incidence of AKI is significantly lower in the colistin cohort than in the PB cohort [10]. There are no related researches on the tubular metabolism of these two kinds of polymyxins and the mechanisms of drug toxicity remain unclear [11]. It is important to note that AKI models in related studies have not been standardized and generally less stable, resulting in notable variations in results. Furthermore, there have not been comparisons between models with different doses and routes of administration. Even though previous studies have assessed whether the models developed AKI within 1–7 days [12, 13], there are no earlier AKI models to predict the early molecular warning systems and potential therapeutic targets for AKI. Therefore, we aimed to explore an earlier and more stable AKI model by measuring GFR and AKI biomarkers in models using different administration routes and doses of polymyxins, which is further validated by histopathological assessments.

AKI is characterized by a decline in kidney function and a reduction in glomerular filtration rate (GFR), accompanied by an accumulation of metabolic waste products such as creatinine and urea in the blood [14]. Serum creatinine (Scr) and blood urea nitrogen (BUN) are the most common clinical biomarkers for identifying and grading AKI. However, these biomarkers have a certain delay. Scr and BUN levels are raised 72 h after the initial kidney injury when kidney function has already decreased by 30% to 40% [15, 16]. Known biomarkers of tubular injury include kidney injury molecule-1 (KIM-1), liver-type fatty acid-binding protein (L-FABP), neutrophil gelatinase-associated lipocalin (NGAL), angiotensinogen (AGT) and osteopontin (OPN). Among these biomarkers, KIM-1, L-FABP, AGT and OPN are expressed by proximal tubules during kidney injury, while NGAL is expressed by distal tubules. Moreover, KIM-1 has been shown to correlate with the severity of histological tubular injury and is considered the most accurate predictor of tubular histopathological changes [17]. The levels of KIM-1 can increase more than 100-fold following the occurrence of nephrotoxicity. L-FABP gene expressions rise during ischemic injury. NGAL has been identified as an early biomarker for AKI, with its mRNA expressions rapidly increasing up to 1000-fold in the thick ascending limb and collecting ducts shortly after the onset of AKI [18]. These biomarkers may play a crucial role in locating tubular injury [19–21].

Although GFR is the gold standard for precise assessment of kidney function, traditional methods of assessing GFR are cumbersome and time-consuming. In addition, serial blood or urine samples are required, causing damage to the experimental animals. A non-invasive, real-time monitoring technique has been developed to circumvent these shortcomings. Such GFR monitoring techniques in mice allow early detection of AKI, which measures kidney function transcutaneously using an optical device and the exogenous kidney marker fluorescein isothiocyanate (FITC)-sinistrin. This technique can measure the elimination kinetics of the marker through the skin to determine kidney function in real-time without the need for plasma or urine collection and surgical catheterization for drug administration. The method has already been validated in different species and successfully applied to several models of kidney pathology [22]. This real-time monitoring technology provides a more accurate and reliable assessment of GFR changes in the same mouse before and after drug infusion. In this study, we combined this real-time GFR monitoring technology with AKI biomarkers (Scr, BUN, NGAL and KIM-1) and kidney histopathology to assess the extent of kidney injury. Our research explored nearly the full range of ethically acceptable and experimentally feasible doses of PB and PE to establish an early and stable polymyxin-induced AKI model, allowing for the identification of earlier biomarkers and conducting earlier clinical detection, intervention and treatment.

## Methods

### Reagents

Polymyxin B was purchased from Shanghai SPH No. 1 Biochemical and Pharmaceutical Co., Ltd. Polymyxin E was purchased from CSPC Ouyi Pharmaceutical Co., Ltd. Nanjing Jiancheng Bioengineering Institute provided Scr test kits. cDNA reverse transcription kit was from Applied Biosystems (Foster City, CA, USA). UltraSYBR Mixture was from Bimake (2× SYBR Green PCR Master Mix, Cat No. B21203).

### Animal model

**Fig. 1:**
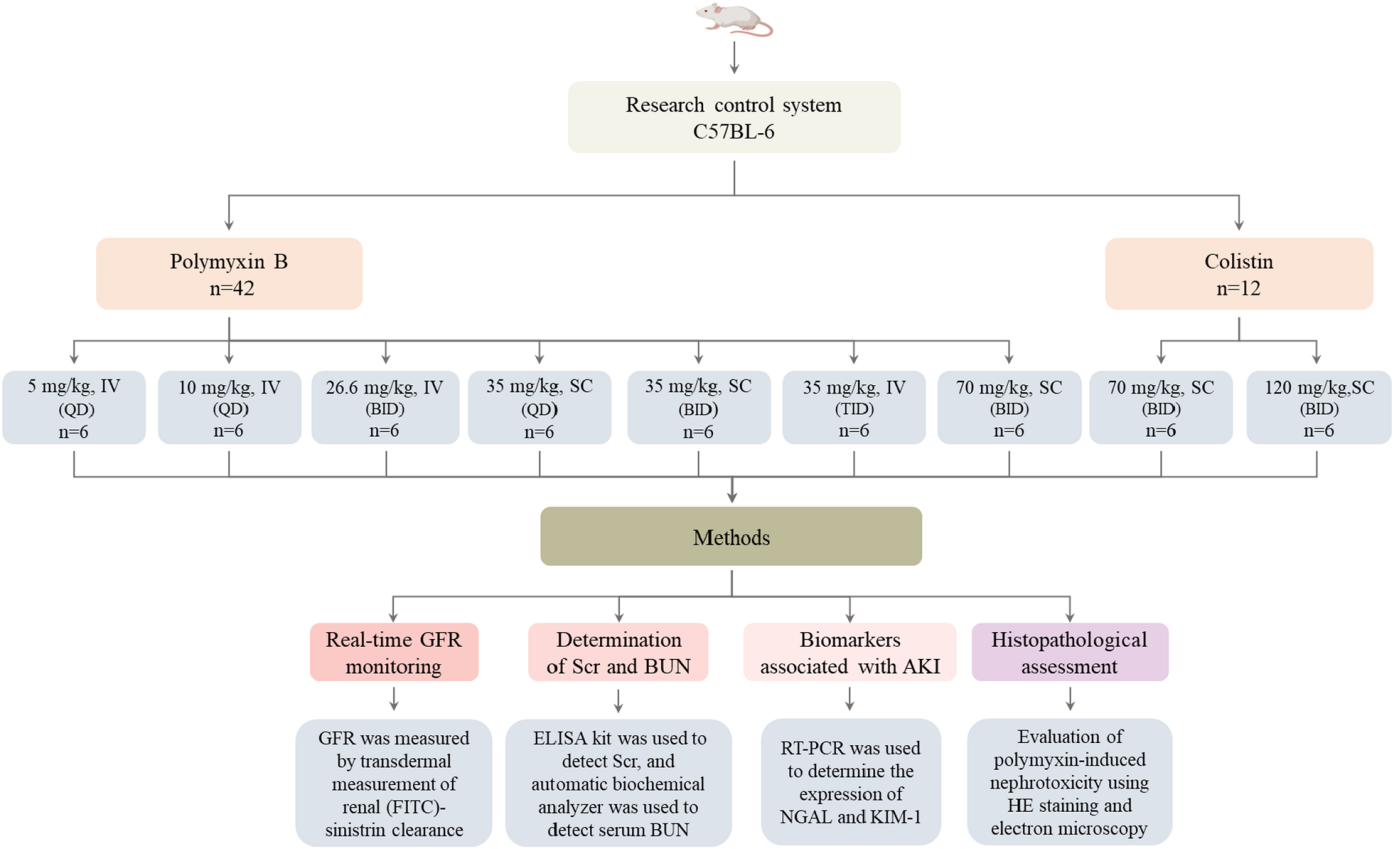
Flowchart of experimental groups and detection methods. All doses shown are total doses divided into one, two, or three injections (denoted as QD, BID, TID). *QD* once daily, *BID* twice daily, *TID* thrice daily, *IV* intravenous, *SC* subcutaneous, *GFR* glomerular filtration rate, *RT-PCR* reverse transcription PCR

In this study, we used a total of 66 male C57BL/6 mice (20–25 g, 6–8 weeks, Beijing Vital River Laboratory Animal Technology Co., Ltd.), which were kept under moderated housing conditions (12-h light/dark cycle, constant temperature of 23 °C, 60% humidity, with adequate food and water). The mice were randomly divided into 11 groups. Each group consisted of six mice. Among the groups, seven were administered PB at different concentrations for 3 consecutive days (four groups via tail vein injection and three via subcutaneous injection). Two groups received PE at different concentrations for 3 consecutive days (both via subcutaneous injection). The remaining two groups served as controls: one group received a tail vein injection, and the other received a subcutaneous injection, both with the same volume of saline as the experimental groups. For the group that received PB at a dose of 35 mg/kg/day subcutaneously once daily, GFR was measured using fluorescence quantification technology before dosing and at 2 h, 4 h, 6 h, 8 h, and 24 h after the 1st dose. (0 h, 2 h, 4 h, 6 h, 8 h, 24 h), 24 h after the 2nd dose (48 h), and 24 h after the 3rd dose (72 h). Biomarkers of kidney injury (Scr, BUN, NGAL and KIM-1) were measured at 2 h, 8 h, 24 h, 48 h, and 72 h. Additionally, histopathology was assessed according to standard criteria at 2 h, 8 h, and 48 h. In the remaining nine experimental groups, GFR was measured at 24, 48, and 72 h, and biomarkers of renal injury were assessed at 72 h. When the predetermined endpoint was reached, the mice were anesthetized by intraperitoneal injection of pentobarbital sodium (1%, 40 mg/kg; Macklin, Shanghai, China) and killed by cervical dislocation. Blood samples were then collected to detect BUN and Scr. After bilateral kidneys were extracted, one was placed in a cryotube and was promptly stored in a − 80 °C freezer, and the other was placed in 4% paraformaldehyde [23]. Every effort was made to reduce the pain experienced by the mice throughout the experiment. We compared the GFR changes in the same mice before and after drug infusion, along with the levels of renal injury biomarkers (Scr, BUN, NGAL, KIM-1) between the control and experimental groups, and performed histopathological assessments of tubular damage, all aimed at determining the occurrence and extent of renal injury in the experimental group. All models and experimental methods in this study are shown in Fig. 1. This experiment was approved by the Animal Management and Welfare Ethics Committee of the First Affiliated Hospital of Harbin Medical University (Approval No. IACUC: 2023022). This animal experiment follows the National Health and Medical Research Council guidelines on using animals for scientific purposes. All dosages adhered to the ethical requirements for animal experiment [24].

### Transcutaneous measurement of GFR

GFR was measured by transcutaneous determination of the clearance rate of protein-tagged FITC-sinistrin. Mice were lightly anesthetized with isoflurane (RWD, flow rate 300 ml/min, concentration 1%) and the hair on the left dorsal kidney area was removed. A fluorescence detector (NIC-Kidney, Mannheim Pharma&Diagnostics, Germany) with a transparent window (1 × 0.5 cm) was affixed to the depilated area using double-sided adhesive tape and adhesive tape [25]. Once the mice regained consciousness and hemodynamic stability, FITC-sinistrin (1 mg/0.1 ml/100 g) was injected via the tail vein. After 60 min, the optical device was removed. The acquired data were exported to a computer for analysis using MediBeacon Studio V2 software (NIC-Kidney, Mannheim Pharma and Diagnostics, Germany).

### Detection of BUN, Scr

Once all samples were collected, serum BUN levels were measured using an automated biochemical analyzer (IDEXX Catalyst One). Plasma samples were analyzed for Scr levels using test kits (creatinine assay kit, Nanjing Jiancheng Bioengineering Institute, Nanjing).

### Determination of KIM-1, NGAL

Kidney tissues were used for total RNA extraction using TRIzol reagent (Invitrogen, San Diego, CA, USA) following the standard protocols. cDNA was synthesized from 1 µg of RNA using the High Capacity cDNA Reverse Transcription Kit. Relative levels of target genes were determined by qPCR using UltraSYBR Mixture. The PCR primer sequences used are listed in Table 1. Transcript expression levels were calculated using the comparative CT method and normalized to actin levels (2−ΔΔCT). In this study, β-actin expression levels did not show statistical differences between groups. The 2−ΔΔCT method was used to calculate the relative gene expression of early kidney injury biomarkers NGAL and KIM-1 [17].

**Table 1:** List of primers for PCR of mice

Primer	Sequence
β-Actin	
Forward	5′-CCCTAAGGCCAACCGTGAA-3′
Reverse	5′-GAGGCATACAGGGA CAACA-3′
NGAL	
Forward	5′-TCTGGGCCTCAAGGATAACA-3′
Reverse	5′-AGACAGGTGGGACCTGACCA-3′
KIM-1	
Forward	5′-GTCTGTATTGTTGTCGAGTGGAG-3′
Reverse	5′-CGTGTGGGAATCTCTGGTTTAAC-3′

### Tubule damage assessment

Kidney tissues were cut into small pieces, dehydrated and embedded in paraffin, then made into paraffin-embedded blocks, sliced and stained with hematoxylin and eosin (HE). The pathological sections were observed under a 400× light microscope. Five fields were randomly selected, and ten renal tubules were selected for each field. The scoring criteria were according to Paller’s method, i.e., apparent dilatation of renal tubules, flattened cells (1 point), tubular type (2 points), exfoliated and necrotic cells in the lumen of renal tubules, but no tubular type and cell debris (1 point), granular degeneration of epithelial cells (1 point), vacuolar degeneration (1 point), nuclear pyknosis (1 point). The score was used to evaluate the degree of injury. SPSS statistical software was used to analyze the Paller score of HE sections in each group, and *t*-test was used to evaluate the significance of the difference between the two groups. One-way ANOVA was used to compare differences across multiple time points [26].

### Statistical analysis

Statistical analyses were performed using Prism 9.5.1 (GraphPad, La Jolla, CA). One-way repeated measures ANOVA was used to analyze various indicators across multiple time points. Paired t-tests were used for comparisons between the two groups. A* P*-value of less than 0.05 was considered statistically significant. Data are presented as mean ± standard error [26].

## Results

### Characteristics of PB groups

#### PB (dosage > 13.3 mg/kg/day intravenously) once daily

When the single intravenous injection of PB exceeded 13.3 mg/kg, mice experienced immediate seizures followed by sudden cardiac arrest and death. This may be related to the neurotoxicity of polymyxin B. A report from John et al. indicated that the administration of high doses of intravenous polymyxin B (3–6 mg/kg/day) are coincident with increased neurotoxicity events in patients [27]. The primary clinical manifestations of neurotoxic adverse reactions to polymyxin antibiotics include seizures, cardiac arrest, and respiratory distress [28, 29].

#### PB (35 mg/kg/day subcutaneously) once daily

**Fig. 2:**
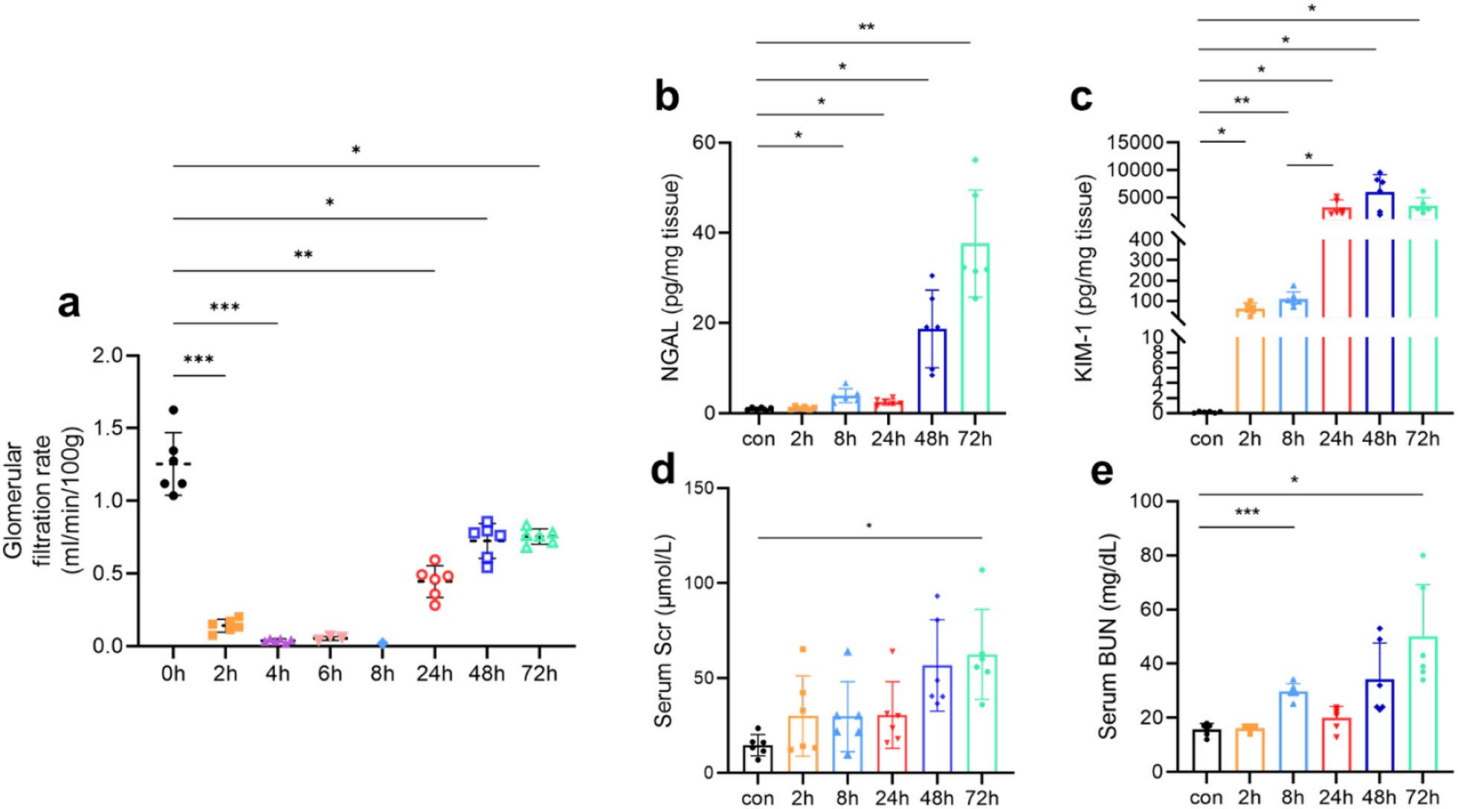
The results of mice receiving PB (35 mg/kg/day subcutaneously) once daily. **a** Glomerular filtration rate (GFR) values in mice at 0, 2, 4, 6, 8, 24, 48, and 72 h after PB injection. **b** Representative NGAL gene expression levels in mice kidneys at 2, 8, 24, 48, and 72 h after PB injection. **c** Representative KIM-1 gene expression levels in mice kidneys at 2, 8, 24, 48, and 72 h after PB injection. **d** Serum creatinine (Scr) levels in mice at 2, 8, 24, 48, and 72 h after PB injection. **e** Blood urea nitrogen (BUN) levels in mice at 2, 8, 24, 48, and 72 h after PB injection

GFR: Fig. 2a shows that GFR decreased gradually after PB injection. At 6 h, GFR was undetectable in 3 out of 7 mice (3/7). At 8 h, GFR was undetectable in 5 out of 7 mice (5/7), indicating almost complete kidney cessation of FITC-sinistrin excretion. The remaining mice exhibited significantly reduced GFR values (*P* < 0.0001). At 24, 48 and 72 h, GFR gradually recovered but remained significantly lower than pre-injection levels (*P* < 0.0001).

AKI damage biomarkers (NGAL and KIM-1): Fig. 2b, c shows that KIM-1 levels increased approximately 100-fold at 2 h compared to the control group (*P* = 0.0148) and further increased at 24 h. The differences among 24, 48, and 72 h were not statistically significant. NGAL expression increased approximately tenfold at 8 h (*P* = 0.0308) and rose to several 100-fold at 48 and 72 h compared to the control group. This trend was consistent with changes in GFR, Scr, BUN and histopathological evaluations.

AKI functional biomarkers (Scr and BUN): Fig. 2d, e shows that BUN levels increased significantly at 8 h (*P* = 0.0007) and 72 h (*P* = 0.0406). The trend of Scr changes was similar to that of BUN, with a significant increase observed at 72 h compared to the control group (*P* = 0.0272).

**Fig. 3:**
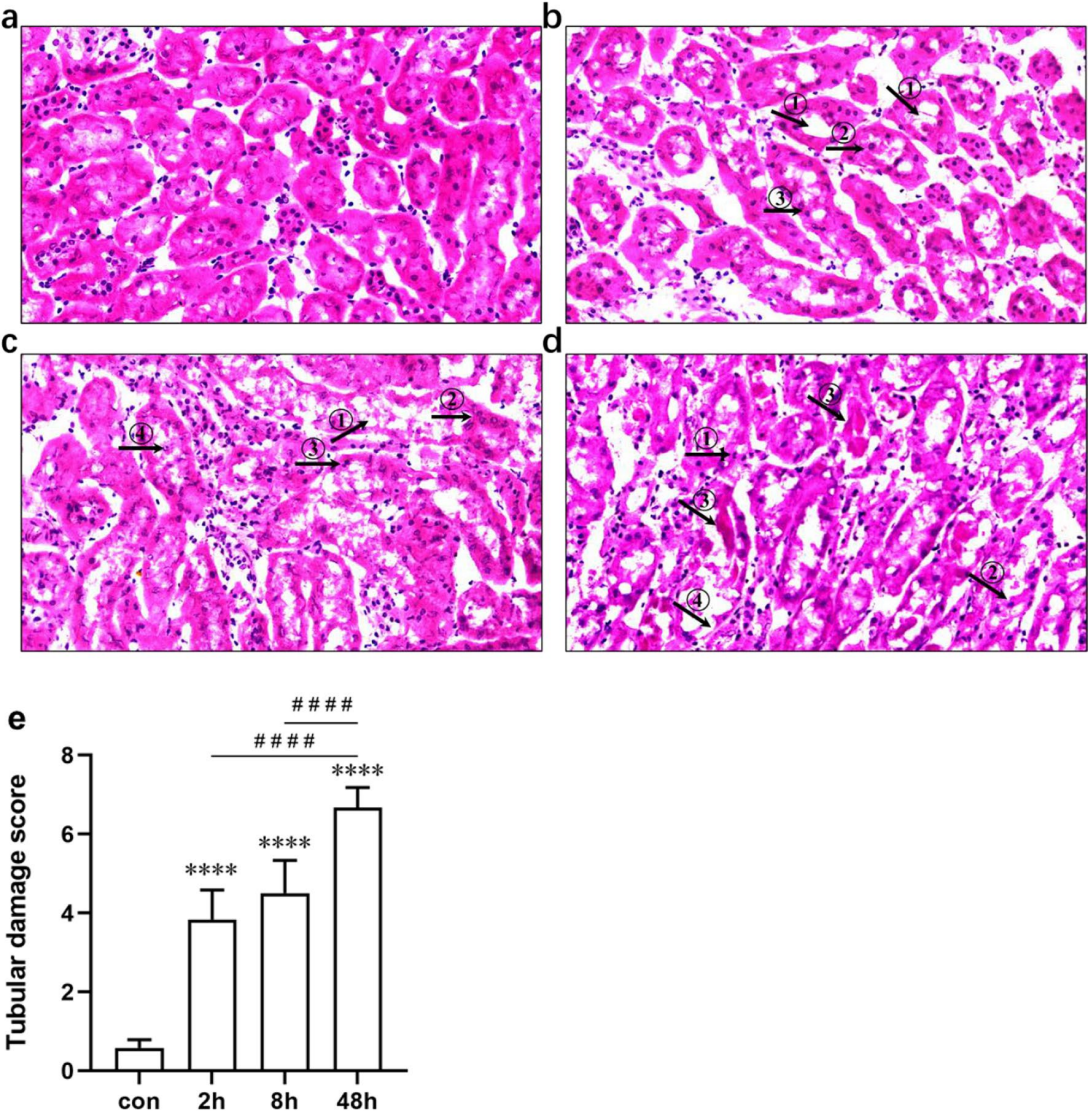
Histopathological evaluation of renal tubular injury was performed at 2, 8, and 48 h in the group receiving PB (35 mg/kg/day subcutaneously) once daily. **a** Histopathological images of kidney tissues from the control group (*n* = 6). **b** Representative photomicrographs of tubular injury at 2 h after PB administration (*n* = 6). ①: obvious expansion of renal tubules. ②: exfoliated and necrotic cells. ③: flattened cells. **c** Representative photomicrographs of tubular injury at 8 h after administration of PB (*n* = 6). ①: vacuolar degeneration of epithelial cells. ②: granular degeneration of epithelial cells. ③: nuclear pyknosis. ④: exfoliated and necrotic cells in the lumen of renal tubules. **d** Representative micrographs of tubular injury almost 24 h after the second dose. ①: nuclear pyknosis of tubular epithelial cells. ②: exfoliated and necrotic cells. ③: tubular type. ④: denuded basement membrane. **e** Quantitative assessment of tubular injury at 2, 8, and 48 h after dosing (*n* = 6). ****P < 0.0001 vs control group. ^####^*P* < 0.0001 vs 48 h group, con: control

Histopathological evaluations: Fig. 3 shows that significant kidney injury occurred as early as 2 h after the first dose, and the injury further worsened at 48 h.

#### PB (70 mg/kg/day subcutaneously) twice daily

Among six mice that received PB (70 mg/kg/day subcutaneously) twice daily for 3 days, four died after 1 day and the remaining two did not survive to the scheduled endpoint. After drug administration, the mice ceased activity and feeding, exhibited body convulsions, and died after 1 day.

This finding may also be related to the neurotoxicity of PB [30]. Previous clinical studies have demonstrated that the neurotoxicity of polymyxins is dose-dependent. Reported neurologic toxicity has been associated with facial and peripheral paresthesias, seizures, and neuromuscular blockade. The last of these usually produces a myasthenia-like clinical syndrome, as well as respiratory failure or apnea due to respiratory muscle paralysis [31]. These symptoms in the mice may be the combined result of the neurotoxic effects of PB, leading to impaired activity and feeding, and ultimately resulting in death.

### Characteristics of PE groups

#### PE (120 mg/kg/day subcutaneously) twice daily

**Fig. 4:**
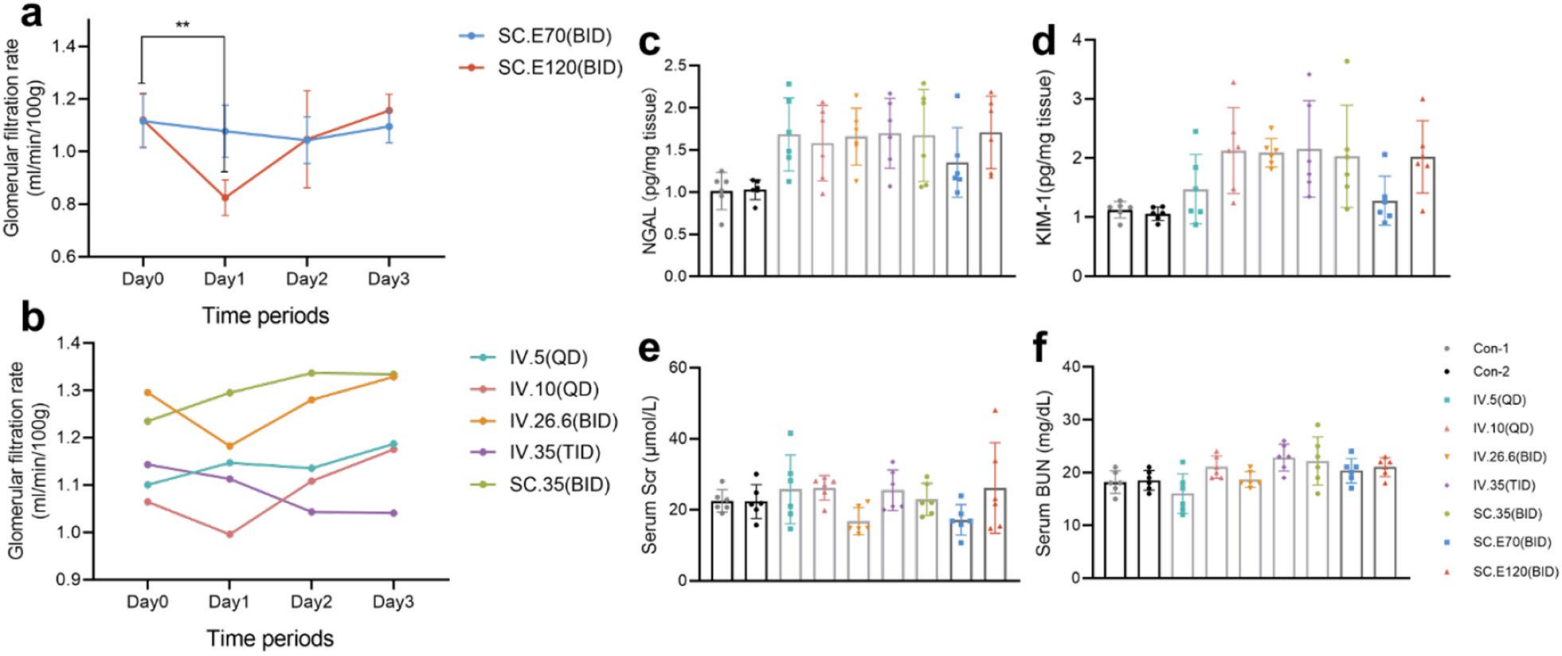
The results of mice receiving PB and PE [except for mice receiving PB (35 mg/kg/day subcutaneously) once daily]. **a** The figure shows the GFR of mice receiving PE at 0, 24, 48, and 72 h. **b** The figure shows the GFR of mice receiving PB at 0, 24, 48, and 72 h. **c**–**f** The figures show the changes in kidney injury biomarkers (NGAL, KIM-1, Scr, and BUN) at 72 h in mice receiving PB and PE. SC.E70: mice receiving PE (70 mg/kg/day subcutaneously); SC.E120: mice receiving PE (120 mg/kg/day subcutaneously); IV.5: mice receiving PB (5 mg/kg/day intravenously); IV.10: mice receiving PB (10 mg/kg/day intravenously); IV.26.6: mice receiving PB (26.6 mg/kg/day intravenously); IV.35: mice receiving PB (35 mg/kg/day intravenously); SC.35 mice receiving PB (35 mg/kg/day subcutaneously); Con-1: control group receiving tail vein saline injection; Con-2: control group receiving subcutaneous injection of saline; QD: once daily; BID: twice daily; TID: thrice daily

GFR: After subcutaneous injection of PE, the GFR of mice decreased significantly at 24 h, but returned to normal at 48 h and 72 h (Fig. 4a).

Scr, BUN, NGAL, and KIM-1: After the post-3rd dose, there were no statistically significant differences in Scr, BUN, NGAL and KIM-1 levels between the experimental and control groups, indicating that the mice did not develop AKI. (Fig. 4c–f).

### Characteristics of other groups

There were no significant changes in GFR, Scr, BUN, NGAL, and KIM-1 levels in these groups; detailed information can be found in Supplementary Material 1.

## Discussion

The experiment included 10 model groups. The results in mice receiving PB (35 mg/kg/day subcutaneously) once daily shows the occurrence of AKI as early as 2 h post-1st dose. The other PB groups did not exhibit significant differences in GFR compared to pre-drug injection over 3 consecutive days. AKI biomarkers (Scr, BUN, NGAL, and KIM-1) were not elevated compared to the control group, indicating the absence of AKI. Although GFR decreased at 24 h in the group receiving PE (120 mg/kg/day subcutaneously) twice daily, it normalized at 48 h, and kidney injury biomarkers and GFR remained unchanged at 72 h compared to the control group, indicating the absence of AKI. The model receiving PE at 70 mg/kg/day subcutaneously twice daily showed no significant differences in GFR at 24 h, 48 h, and 72 h compared to the pre-dose administration, and kidney injury biomarkers were not elevated at 72 h, indicating the absence of AKI in this group.

In previous studies, the route of administration, dosage, frequency, and total duration of PB administration were inconsistent. One study utilized 5 mg/kg/day, intravenous injection, once daily for 7 days, and the occurrence of AKI in mice was determined by measuring Scr and BUN levels and histopathological evaluation [25]. Another study used subcutaneous injection, and mice received PB 70 mg/kg/day twice daily for 3.5 consecutive days, and the occurrence of AKI in mice was determined by histopathological evaluation [26]. In contrast to the previous studies, our experiment investigated and compared several mouse models with different drug concentrations, administration frequencies, and routes, and identified a more stable and earlier mouse AKI model induced by polymyxins.

Due to various limiting mechanisms, early kidney injury biomarkers only showed significant increases only in certain areas of tubular damage. The damage sites and mechanisms induced by different factors were diverse, with related researches to be completed, indicating that the changes in known early kidney injury biomarkers may not be consistent with the actual extent of tubular damage. Notably, the non-invasive and real-time monitoring of GFR avoids these shortcomings and can detect kidney injury earlier and more accurately. In our study, GFR showed a significant decline as early as 2 h post-1st dose, which was a phenomenon not reported in previous studies. Simultaneously, we measured changes in the kidney injury biomarkers KIM-1 and NGAL at 2 h. NGAL did not increase, but KIM-1 had already increased by approximately 100-fold, suggesting that the injury pattern of KIM-1 plays an essential role in PB-induced AKI. The results were consistent with a previous study, and showed that KIM-1 was closely associated with polymyxin-induced AKI and increased significantly on days 1, 2, and 3 after PB administration [32].

We compared mouse models that received PB at the same dose and route (35 mg/kg/day subcutaneously) but at different frequencies (QD, BID, TID). The results showed that mice in the QD group exhibited more severe kidney injury compared to the BID and TID groups. This finding was consistent with previous studies by Wallace et al. [33] and Okoduwa et al. [34]. The increased severity of kidney injury in the QD group may be due to the extended dosing interval leading to reduced renal clearance of PB, resulting in higher accumulation of PB in proximal tubular cells. Given the dose-dependent nephrotoxicity of PB and thus the frequency of QD caused more severe kidney injury.

We also compared the nephrotoxicity of PB and PE. The study by Zavascki et al. [9] showed that patients treated with PE had a higher incidence of AKI than those treated with PB. However, Zhang et al. [10] found that PE had less toxicity and a significantly lower incidence of AKI than PB. Our results indicated that PE had less toxicity than PB, which was consistent with those of Zhang et al. The group receiving PE (70 mg/kg/day subcutaneously) twice daily showed no significant differences in GFR or kidney injury biomarkers compared with the control group. The results of the group receiving PE (120 mg/kg/day subcutaneously) twice daily suggested that PE caused mild kidney dysfunction at 24 h, and kidney function recovered autonomously at 48 h. The normal levels of AKI biomarkers at 72 h support our hypothesis that PE-induced kidney dysfunction is reversible. The experiment was designed to identify a more stable polymyxin-induced AKI model by comparing different models. During this process, we observed some unexplained events, such as mice experiencing seizures followed by cardiac arrest when administered PB by tail vein injection at doses higher than 13.3 mg/kg. We speculated that PB may affect the hemodynamics of the mice, but we did not monitor their hemodynamics.

This work also has some limitations. The main limitation of this study was the lack of an in-depth investigation of the mechanisms by which PB induces AKI. Due to species differences, there are significant physiological and pharmacokinetic discrepancies between mice and humans. The metabolic pathways and toxic mechanisms of polymyxin in mice may differ from those in humans, making it challenging for the mouse model to fully replicate the effects of polymyxin on the human kidney. This difference may limit the generalizability of the study’s findings. Our research used healthy mice as models; however, in clinical practice, patients exposed to polymyxin are often critically ill, receiving life-sustaining therapies, and suffering from multiple organ dysfunction. These clinical factors can significantly influence how polymyxin affects the kidneys, potentially leading the healthy mouse model to underestimate the nephrotoxic risk of polymyxin in critically ill patients. In this experiment, mice were treated with high doses and short-term exposure to rapidly induce AKI. However, in clinical settings, patients may receive lower doses but prolonged exposure, which could result in different toxicity profiles and pathological changes. Therefore, the differences between experimental conditions and clinical applications may affect the relevance of the results to real-world clinical scenarios.

Overall, we reported a 2-h PB-induced AKI mouse model for the first time. In subsequent mechanistic studies, it may be possible to identify different metabolic signaling pathways and their key molecular targets. Our future research will focus on exploring these mechanisms using metabolomics and proteomics analyses, which will aid in the early prevention of AKI, mitigate disease damage, improve patient outcomes, and ultimately reduce the global burden of AKI.

## Conclusion

Our study established an early and stable polymyxin-induced AKI model for the first time, facilitating research into the mechanisms of early kidney injury and early prevention strategies for AKI. In addition, our results showed that PE has less toxicity than PB, and mice receiving the same dose of PB in the QD group had more severe kidney injury than the BID and TID groups.

## Supplementary Information

Supplementary Material 1.

Supplementary Material 2.

## Data Availability

All data generated or analyzed during this study are included in this published article and Supplementary Material 2.

## Abbreviations

AKI: Acute kidney injury
PB: Polymyxin
BPE: Polymyxin E
KIM-1: Kidney injury molecule-1
NGAL: Neutrophil gelatinase-associated lipocalin
Scr: Serum creatinine
BUN: Blood urea nitrogen
QD: Once daily
BID: Twice daily
TID: Thrice daily
FITC: Fluorescein isothiocyanate
IV: Intravenous
SC: Subcutaneous
