# Supplementary material for: An early and stable mouse model of polymyxin-induced acute kidney injury
Source: Intensive Care Med Exp. 2024 Oct 1;12:88. doi: 10.1186/s40635-024-00667-y (PMC11445218; doi:10.1186/s40635-024-00667-y)
Supplement: Supplementary file 1 — Supplementary Material 1. [file 40635_2024_667_MOESM1_ESM.docx]

# Characteristics of Other Groups

### PB (5 mg/kg/day intravenously) once daily

**GFR:** After intravenous injection of PB, there were no significant differences in GFR at 24, 48, and 72 h compared to pre-dosing (**Figure 3b**).

**Scr, BUN, NGAL, and KIM-1:** The levels of AKI biomarkers at 72 h showed no increase compared to the control group, indicating that the mice did not develop AKI (**Figure 3c,** **3d,** **3e,** and **3f**).

### PB (10 mg/kg/day intravenously) once daily

**GFR:** Following intravenous injection of PB, no significant differences in GFR were observed at 24, 48, and 72 h compared to predrug (**Figure 3b**).

**Scr, BUN, NGAL, and KIM-1:** There was no increase in the levels of AKI biomarkers at 72 h compared to the control group, indicating that the mice did not develop AKI (**Figure 3c,** **3d,** **3e,** and **3f**).

### PB (26.6 mg/kg/day intravenously) twice daily

**GFR:** There were no significant changes in the GFR of the mice at 24, 48, and 72 h post treatment compared to the pre-drug intervention (**Figure 3b**).

**Scr, BUN, NGAL, and KIM-1:** There were no statistically significant differences in the levels of kidney injury biomarkers between the intervention and the control groups at 72 h (**Figure 3c,** **3d,** **3e,** and **3f**).

### PB (35 mg/kg/day subcutaneously) twice daily

**GFR:** This group of mice was monitored continuously for three consecutive days. The results indicated no significant change in GFR after the intervention compared to the pre-intervention levels (**Figure 3b**).

**Scr, BUN, NGAL, and KIM-1:** There was no obvious increase at 72 h in the levels of AKI biomarkers compared to the control group (**Figure 3c,** **3d,** **3e,** and **3f**).

### PB (35 mg/kg/day Intravenously) thrice daily

**GFR:** There was no significant change in GFR for three consecutive days compared to pre-intervention (**Figure 3b**).

**Scr, BUN, NGAL, and KIM-1:** After administration, the levels of kidney injury biomarkers did not show any significant changes in the intervention group compared to the control group (**Figure 3c,** **3d,** **3e,** and **3f**).

### PE (70 mg/kg/day subcutaneously) twice daily

**GFR:** After subcutaneous injection of PE, GFRs at 24, 48, and 72 h were not significantly different from the pre-injection levels (**Figure 3a**).

**Scr, BUN, NGAL, and KIM-1:** The levels of AKI biomarkers in the experimental group did not increase at 72 h compared to the control group. (**Figure 3c,** **3d,** **3e,** and **3f**).
